# Are We Ready? A Qualitative Study on the Knowledge Status and Gaps of Volunteers in Inclusive Disaster Management

**DOI:** 10.3390/healthcare13202581

**Published:** 2025-10-14

**Authors:** Erkan Kurnaz, Elçin Yüksel-Akgün, Tezcan Çavuşoğlu

**Affiliations:** 1 Computer Research and Application Center, Anadolu University, Eskişehir 26470, Türkiye; 2 Department of Special Education, Faculty of Education, Anadolu University, Eskişehir 26470, Türkiye; elcinogut@anadolu.edu.tr; 3 Department of Special Education, Dede Korkut Faculty of Education, Kafkas University, Kars 36000, Türkiye; cavusoglutezcan@gmail.com

**Keywords:** inclusive disaster management, search and rescue, special needs, disability, communication barriers, volunteer training

## Abstract

**Background/Objectives:** Inclusive disaster management is increasingly recognized as essential for mitigating the heightened vulnerabilities faced by individuals with special needs during crises. This study aimed to examine the knowledge, perceptions, and practices of Search and Rescue Non-Governmental Organization (SAR-NGO) volunteers regarding the inclusion of individuals with special needs in disaster and emergency response scenarios. **Methods:** This qualitative case study was conducted in Eskişehir, Türkiye, involving 20 accredited SAR-NGO volunteers selected through criterion sampling. Data were collected through semi-structured interviews, document analysis, and reflective diaries by the researcher. The study explored the conceptualizations and operational practices of volunteers concerning inclusivity in disaster settings. **Results:** Findings indicate that SAR-NGO volunteers primarily associate special needs with visible physical and sensory impairments, demonstrating limited awareness of cognitive and invisible disabilities. Volunteers reported difficulties in identifying individuals with special needs during emergencies and encountered substantial communication barriers due to the lack of alternative communication tools and insufficient training. Despite receiving extensive technical training in search and rescue operations, participants revealed a significant lack of formal education on inclusive practices. **Conclusions:** Effective inclusive disaster response necessitates not only technical proficiency but also structured training in disability awareness, accessible communication, and inclusive intervention strategies. The study recommends revising volunteer training curricula to integrate participatory models involving individuals with disabilities and expanding inclusive disaster research across various regions. These findings highlight critical gaps in current disaster response systems and underscore the need for systemic reforms to achieve inclusive resilience.

## 1. Introduction

Disasters, whether natural or human-made, have significant physical, psychological, and social effects on communities [1,2,3,4]. These effects are unevenly distributed, as vulnerable populations face increased risks in evacuation, shelter, healthcare, and communication [5,6]. This underscores the ethical and practical importance of inclusive disaster management strategies that prioritize accessibility and fairness [5]. International frameworks, such as the Sendai Framework and the United Nations Office for Disaster Risk Reduction (UNDRR), focus on reducing social inequalities and protecting vulnerable groups to enhance disaster resilience [7,8,9,10] Building on these frameworks, the Convention on the Rights of Persons with Disabilities (CRPD) highlights inclusion as a fundamental principle [11,12,13,14]. However, research indicates that implementation often falls short at the local level, especially in low- and middle-income countries, leaving people with disabilities inadequately protected during emergencies [15,16,17,18,19].

Existing evidence indicates that people with disabilities encounter significant barriers in preparedness, evacuation, shelter, and healthcare due to physical, cognitive, and sensory challenges [5,20,21]. For example, individuals with hearing impairments struggle to access emergency alerts, those with visual impairments face evacuation difficulties, and people with mobility restrictions experience major obstacles during rapid response efforts [7,20,22,23,24,25,26]. Despite rising awareness of these risks, efforts toward inclusive disaster management remain fragmented and inconsistent [11,12,27]. True inclusion necessitates not only policy improvements [8] but also structural reforms in infrastructure [1,28] and enhanced capacity within civil society organizations (CSOs) [29,30]. Research emphasizes that involving people with disabilities as active participants in disaster planning, rather than just passive aid recipients, improves both ethical commitments and operational effectiveness [5,11,12,31,32,33,34].

In Türkiye, research shows that disaster response systems do not adequately meet the needs of people with disabilities [35,36,37,38,39]. For example, the 2023 Kahramanmaraş Earthquakes revealed significant gaps: people with hearing impairments did not receive timely alerts, those with visual impairments faced serious evacuation difficulties, and individuals with mobility challenges had limited access to shelters [37,39,40,41,42]. These problems arise from both infrastructural deficiencies and insufficient preparedness among search and rescue (SAR) teams [41,42,43,44]. Volunteer-led search and rescue non-governmental organizations (SAR-NGOs) play a vital role in disaster response, but they often lack the specialized training and resources needed for inclusive practices [5,12,45,46,47,48,49,50]. Effective emergency response requires not only technical skills but also training in accessible evacuation procedures, inclusive communication, and emergency standards [5,21,49,51,52]. Despite their importance, these skills are limited within Türkiye’s disaster management system, where SAR-NGO involvement is hindered by poor coordination, lack of training, and inaccessible infrastructure [43,53,54,55].

To promote inclusive disaster management, it is crucial to examine the preparedness and perspectives of SAR-NGO volunteers. Current research highlights a gap: few studies explore volunteers’ knowledge and practices in supporting people with disabilities during disasters [56,57,58,59]. This gap creates risks, as untrained teams might unintentionally endanger individuals with special needs during emergencies [5,60]. Addressing this involves three main steps: (a) specialized training on communication and evacuation of people with disabilities [12,25,47,49]; (b) accessible disaster planning that considers diverse physical, cognitive, and sensory needs [5,61]; and (c) active involvement of people with disabilities in planning and decision-making processes [11,34,62,63,64,65]. In this context, this study aims to assess the knowledge and practices of SAR-NGO volunteers regarding inclusive disaster management in Türkiye. Specifically, it investigates what information volunteers possess, how applicable it is in real situations, and the adequacy of current training programs. The findings are expected to guide improvements in training curricula and inform policies to foster more inclusive and resilient disaster management.

## 2. Method

### 2.1. Research Design

This study employed a qualitative case study approach. It explored the practices of search and rescue organizations in Eskişehir, Türkiye, to understand how they support individuals with special needs during disasters and emergencies. Eskişehir was selected because of its high number of NGOs, disaster risk exposure, and the researchers’ access to local associations. Due to the limited number of studies in Türkiye addressing the involvement of individuals with special needs in disasters and emergencies, particularly in search and rescue efforts, this research was conducted as a regional single-case study focused on description and exploration [66,67,68,69,70].

### 2.2. Participants

Participants were selected using criterion sampling [71]. The inclusion criteria were: (a) being an active member of an accredited search and rescue association in Eskişehir Province; (b) having experience intervening with individuals with special needs during disasters and emergencies; and (c) willingness to voluntarily participate in the study. According to the Association Information System of the Ministry of Internal Affairs of the Republic of Türkiye, five accredited associations in Eskişehir met these criteria. Researchers formally contacted each association by phone and email, explained the study objectives, and obtained organizational approval before approaching individual members. Volunteers were invited through official association channels, and those who expressed interest received information sheets outlining the study aims, procedures, and ethical safeguards. Written informed consent was obtained from all participants, who were assured that their involvement was voluntary, confidential, and would not affect their organizational role or standing. Interviews were conducted in quiet, accessible spaces, including association meeting rooms and university research facilities, each lasting between 45 and 75 min.

A total of 20 participants who met the inclusion criteria were recruited (Table 1). Although the associations were based in Eskişehir, many participants had also contributed to disaster responses at the national level, especially during the 6 February 2023 Kahramanmaraş earthquakes, which several participants mentioned during the interviews. Eskişehir was selected as the study site because it has a high density of non-governmental organizations (NGOs), exposure to disaster risks, and the researchers’ institutional access to local groups, which made recruitment and in-depth engagement with volunteers easier.

### 2.3. Researchers

All of the researchers have a postgraduate degree in special education. Two researchers have a PhD, and one researcher is continuing his doctorate. They have also published research in international, peer-reviewed journals on qualitative research methods related to the experiences of various professionals intervening with individuals with special needs. In addition, two researchers have participated in national and international funded projects on disaster management of individuals with special needs.

### 2.4. Data Collection Process

Permission for the study was obtained from the Anadolu University Social Sciences and Humanities Research and Publication Ethics Committee (Permission number: 682.035). A semi-structured interview form was prepared after reviewing relevant literature and initially included 15 questions. The draft was evaluated by six experts with doctoral degrees in special education, qualitative research, and disaster-emergency response. Based on their feedback, the final form consisted of 13 questions focusing on issues such as communication with individuals with special needs during disasters and providing first aid services. Semi-structured interviews were conducted face-to-face by the third researcher between 2 September 2024 and 31 October 2024. Sessions took place either in participants’ workplaces or in neutral NGO offices, depending on their preference, to ensure confidentiality and comfort. Interviews lasted between 50 and 115 min.

Besides interviews, data were gathered through document analysis and researcher diaries. The documents included association statutes, volunteer training records, and materials used during disaster response [72,73]. These were organized and analyzed to support the interview results. The researchers also kept reflective diaries throughout the study [74], which featured observations of research settings and unstructured conversations with participants. These diaries added an extra layer of data, enriching the understanding of volunteers’ experiences and practices.

### 2.5. Data Analysis

The data were analysed using reflective thematic analysis [59]. All sources—interviews, researcher diaries, and documents—were coded following the same procedure. Three researchers independently coded subsets of the transcripts, then compared their coding and discussed discrepancies until consensus was reached. To ensure accuracy, interview recordings were repeatedly listened to, transcribed verbatim, and reviewed by an independent qualitative researcher before being consolidated into a single dataset. Researcher diaries and documents collected during the study were integrated into this dataset, which was subsequently analysed in NVivo Plus.

Reflexivity was considered throughout the analysis. Each researcher kept reflective notes documenting their assumptions, observations, and potential biases, which were discussed during team meetings to minimize individual influence on coding decisions. After initial coding, the framework was reviewed by an external qualitative research expert, who provided feedback and confirmed the coding structure. Codes were then organised into sub-themes and synthesized into three main themes and four sub-themes through collaborative discussion. In addition, the organizational charts and bylaws of SAR-NGOs were reviewed to contextualize the findings. All documents are reported in the results section with identifiers and access dates (e.g., documents, 23122024). Using multiple data sources and incorporating reflexivity enhanced the credibility and depth of the analysis.

### 2.6. Trustworthiness

Several strategies were implemented to ensure the trustworthiness of the study, addressing credibility, transferability, dependability, and confirmability [75,76]. Credibility was strengthened by using multiple data sources—including interviews, document analysis, and researcher diaries—and by presenting preliminary findings to participants for validation. Three volunteers reviewed coded transcripts and discussed the accuracy of interpretations during follow-up meetings, with their feedback incorporated into theme refinement. Transferability was supported by offering detailed descriptions of participants, settings, and procedures, enabling readers to assess the applicability of the findings to other contexts. Dependability and confirmability were reinforced by storing data with multiple recording systems, presenting participants’ views under pseudonyms to protect anonymity, and having transcripts and coded results reviewed by field experts. To further enhance credibility, additional member checks were conducted with participants from different associations and age groups, whose confirmatory remarks helped refine the themes without altering the data’s core meaning. Finally, reflexivity was addressed through reflective diaries, independent coding checks, and member validation, which helped reduce potential interpretive bias stemming from the researchers’ backgrounds in special education.

## 3. Findings

**Note:** In the following section, participants’ direct quotes are presented verbatim. Some terms (e.g., ‘mental retardation,’ ‘crippled,’ ‘these people’) are outdated or othering. They are included here solely to preserve the authenticity of participant voices; the authors do not endorse these terms.

Rich and detailed descriptions were included in the presentation of the findings as a natural requirement of trustworthiness. The thickness and thinness of the relational lines in the code maps were used to express the intensity of opinions on the relevant codes. The participants’ statements were placed in quotation marks to support the narrative. Additionally, data visualizations were used to support the presentation of the findings. At certain points, non-inclusive participant expressions such as “disability” and “impairment” were presented to strengthen the narrative and support credibility. Moreover, the themes and sub-themes were titled using the participants’ statements to enhance the narrative.

**Theme 1: Identification and Perception of Individuals with Special Needs: “You know, a person with special needs may have a physical disability. Not being able to move, basically.”**

Volunteers often defined individuals with special needs in terms of functional limitations, focusing on challenges in communication, mobility, and daily self-care. Their descriptions reflected a reliance on visible characteristics and everyday observations rather than professional knowledge.

**Sub-theme 1: General Characteristics of Individuals Requiring Specialized Care**

Many participants described individuals with special needs as “different” from society because they could not perform certain tasks independently. Communication was a common reference point: “*They cannot express themselves verbally. At the simplest, people who cannot express their wishes like us*” (Orhan). Others emphasized dependence on additional support: “*These people want different and special attention. I mean, most of them cannot speak like us*” (Participant). Mobility limitations were also central: “*Mostly they cannot move independently. So they have to use wheelchairs or crutches*” (Anıl). Such views were extended to self-care and daily activities: “*At the simplest level, these people cannot feed themselves. For example, I had a neighbour… he had difficulty even taking bread from the cupboard and eating it*” (Yusuf). A few participants also associated special needs with illiteracy, noting difficulties in reading or understanding written instructions: “*We cannot communicate with most of them, even in writing*” (Musa).

**Sub-theme 2: Perceptions of Physical, Mental, and Sensory Disabilities**

Several participants equated special needs directly with disability or illness. “*It means special needs. It means people who have difficulties due to disability or illness*” (Metin). Another explained: “*As you know, people with physical, mental, or visual impairments. Some of them also have a disease. Like cerebral palsy*” (Yusuf). However, participants admitted their knowledge was limited, based mainly on personal encounters or occasional training exposure. “*Honestly, I don’t have much knowledge. I know as much as I have encountered. There are blind individuals, and others with physical disabilities*” (Yusuf). “*We received a few trainings and I noticed individuals with mental retardation there. Of course, we encounter them in daily life. I see people using wheelchairs, that’s it*” (Emre).

Overall, participants’ descriptions revealed a narrow and often outdated understanding, with frequent use of culturally rooted or non-inclusive expressions in quotations. While visible impairments such as physical, hearing, and visual disabilities were commonly mentioned, less visible conditions such as autism or dementia were rarely acknowledged. This limited perspective reinforces stereotypes and risks overlooking individuals with invisible disabilities, leading to inadequate or delayed interventions in disaster contexts.

**Theme 2: Challenges in the Response Process and Communication Barriers: “This is no joke. So, while trying to save someone, you may cause them to lose their life.”**

SAR-NGO volunteers emphasized that search and rescue requires a high degree of expertise and continuous training. While many had completed accredited modules in evacuation, surface search, or urban rescue, they admitted that these programs rarely addressed the needs of individuals with disabilities. As one participant explained: “*We completed the surface search training just a month ago. Every emergency has its own specific response. We are trying to prepare as much as we can*” (Anıl). Yet volunteers acknowledged significant limitations when adapting interventions for people with special needs. Musa reflected: “*Of course, we are not perfect. Especially when it comes to individuals with special needs.*” Yusuf further highlighted the stakes: “*This is no joke. While you are trying to save someone, you may cause them to lose their life. Even your simplest mistake can cause someone to lose a limb for life.*”

**Sub-theme 1: Challenges in Identifying Individuals and Insufficient Information**

A recurring problem was difficulty recognizing individuals with special needs during disaster operations, especially when disabilities were not immediately visible. “*If the person does not have a device they use or a visible loss on their body, we have a very difficult time understanding*” (Murat). Another added: “*We can directly understand the hearing impaired, the physically disabled. But if they are mentally disabled, how can you understand them?*” (Anıl).

Volunteers also reported working with little or no background information about individuals during large-scale emergencies. “*Most of the time, we go uninformed… In general, we do not have information about the people in the area we intervene”* (Eray). Some shared experiences from the 2023 earthquakes, describing the difficulty of identifying survivors with hearing impairments trapped under rubble without prior knowledge (Metin).

**Sub-theme 2: Communication Limitations and Professional Deficiencies**

Volunteers consistently cited communication barriers as one of their most serious challenges. When individuals could not communicate verbally, participants often felt powerless. “*If the person I meet cannot speak, we have a big problem. Because I don’t know how to communicate in any other way, I know it’s my fault*” (Musa). Another explained: “*We went to a disappearance case. The person was mentally challenged… he didn’t speak. We had to wait until the relatives came*” (Anıl).

Most associations lacked staff trained in sign language or tools such as communication charts or Braille boards. “*First of all, I don’t know sign language, and I don’t have a tool that will enable me to communicate. Moreover, even if I did, I did not receive any training on how to use them*” (Musa). Aydın described a case during the Adıyaman earthquake where rescuers could not triage a non-verbal individual: “*If there were someone in the team who could sign, our job would have been very easy.*”

Only one participant reported access to alternative methods: “*We received support from professors at the university… We have a triage chart, and a friend of ours was trained in sign language*” (Emre). This exception highlighted that such capacities often depended on individual efforts rather than systematic training.”.

**Theme 3: Inadequacy in Service Delivery and Field Experiences: “We encounter disabled individuals in almost every case we visit. However, it cannot be said that we provide adequate services.”**

SAR-NGO volunteers reported that they encountered people with special needs in almost every disaster and emergency they responded to, from avalanches to industrial fires. Despite this frequency, they admitted feeling limited in their capacity to provide interventions tailored to these individuals. As one participant summarized: “*We encounter people with disabilities in almost every case we visit. However, it cannot be said that we provide adequate services.*”

**Sub-theme 1: Awareness of Deficiencies in Service Delivery**

Volunteers consistently emphasized that their training—both at the national and international levels—did not include content on inclusive disaster management. “*Of course, we are not perfect. Especially when it comes to individuals with special needs. However, we have not seen any content related to this in any training we have received so far”* (Metin). Aydın echoed this gap: “*Five people from our association went abroad for accreditation, and there was nothing about inclusive disaster management in the content explained to us there.*”

This lack of training was seen as directly affecting field practice. “*We are responsible not only for rescuing the person but also for delivering them to health personnel. Without vital information, mistakes can happen that may cost lives*” (Yusuf). Another volunteer reflected on the risks of inexperience: “*We rescued a mentally disabled individual. However, we could not communicate with him, so we waited until his relatives arrived. Luckily, it did not end badly. But what if he had a hidden problem we couldn’t see?*” (Anıl).

Beyond immediate interventions, volunteers pointed out the lack of inclusive content in their preventive work, like disaster awareness training. “*We go to schools. I see children with special needs. These children need disaster training. Maybe their teachers are retelling it, but we should be able to inform the teachers as well*” (Musa). Likewise, workplace trainings often involved contact with people with disabilities but without specific adaptations.

Volunteers also noted that much of their knowledge came from personal encounters rather than systematic preparation. “*I know as much as my own experience. For example, I have never met an individual with autism*” (Veli). Efforts to self-learn were often unsuccessful due to a lack of accessible resources. “*I tried to research on the internet, but I could not find any resources directly focusing on search and rescue and special needs. As far as I searched, there is no book written in Turkish about this*” (Eray).

This sub-theme highlights that the absence of formal training and limited self-learning opportunities result in fragmented and insufficient service delivery for people with disabilities.

**Sub-theme 2: Experiences of Engaging with Individuals in Rural Areas**

Volunteers reported that encounters with people with disabilities were common across disaster contexts, but particularly in missing-person cases and fires. “*When I started volunteering, I didn’t expect this. But in every incident we respond to, I encounter someone with special needs or their relatives. This is normal for us*” (Yusuf). Another added: “*Most of the reports we receive are about missing persons. We often encounter elderly individuals with disabilities*” (Metin).

Industrial and forest fires were frequently mentioned as contexts where volunteers met people with disabilities. “*In a workplace fire, one person was blind and another had a disability. I evacuated both*” (Bora). In rural areas, forest fires posed significant risks due to the high number of elderly or disabled residents. “*For example, in the fire in Datça, there were many villages in the forest. We encountered a large number of elderly or young individuals with disabilities in these areas. It’s always like this in rural settlements around big cities*” (Aylin).

This sub-theme illustrates that people with disabilities are regularly encountered in diverse emergency settings. However, the lack of disability-specific training and planning creates difficulties in providing appropriate and timely support, especially in rural and fire-prone areas.

## 4. Discussion

This study explored how SAR-NGO volunteers in Eskişehir perceive, recognize, and assist individuals with special needs during disasters and emergencies. Instead of just restating the results, the discussion interprets the underlying patterns: volunteers’ perceptions are limited by stereotypes and organizational shortcomings, which together restrict inclusive practices and pose practical and ethical challenges.

Volunteers often equate “special needs” with visible impairments and limitations in independent living. This reflects deficit-focused views in disaster management that see people with disabilities as passive recipients instead of active participants [47]. When recognition relies on visible signs, less visible conditions (e.g., autism, dementia, psychosocial disabilities) can be overlooked, leading to delayed or incorrect interventions. From a sociocultural perspective, this mirrors prevailing ideas of disability as dependency rather than diversity, a view supported in previous research [7,10,22,25].

The language used in interviews—such as “crippled,” “mental retardation,” or “these people”—further demonstrates how cultural framings influence practice. Such terminology contradicts person-first, rights-based approaches and is more than just stylistic; it shows how organizational training and broader social narratives normalize othering. Previous research notes that responders often lack clear guidance on respectful, rights-based language, allowing exclusionary terms to persist in training and field communication [22,29]. The current data support this: outdated language in participants’ accounts suggests that discourse itself can reinforce barriers to inclusion.

Operationally, volunteers reported ongoing difficulties in identifying and communicating with individuals with special needs when no prior information was available. This pattern suggests that the lack of systematic information-sharing mechanisms acts as a structural barrier rather than just an operational oversight. Effective crisis response relies on personalized, context-aware, and information-based decision-making [6]; yet participants described a lack of the infrastructure necessary for such decisions [8,77,78,79,80,81,82,83]. Communication barriers worsened this issue: limited training in alternative methods (e.g., sign language, symbol boards, visually supported triage) and the absence of related equipment hindered timely and appropriate support [21,49,51,84]. These shortcomings confirm that accessibility is both a human right and a technical necessity for coordinated response [14,63,85,86,87], and that inclusive capacity is fundamental—not peripheral—to accurate needs assessment and intervention [34,52,87,88,89,90].

A key issue revolves around training and accreditation. Participants consistently noted that neither national programs nor international accreditation processes included systematic modules on disability inclusion (e.g., “we have not seen any content related to this in any training we have received so far”). Because these responses were similar across organizations, they should not be seen as isolated oversights but as a recurring pattern. Previous critiques of international standards point out similar gaps [29,45,77,90,91,92,93,94,95]. Therefore, calling this a “structural failure” or “systemic disregard” is supported by the data: when inclusion is missing from accreditation frameworks, exclusionary practices are quietly accepted at national and local levels, perpetuating cycles of unpreparedness.

Overall, the findings suggest that practice limitations are shaped by both sociocultural ideas (which influence how disability is understood and discussed) and organizational structures (which determine what is taught, resourced, and evaluated). Addressing these issues, therefore, requires system-wide change; specific recommendations are provided in the Conclusion.

### Limitations

This study was conducted exclusively with SAR-NGO volunteers active in Eskişehir, which limits how well the findings can be applied to other regions with different risk profiles, organizational structures, or volunteer cultures. Participation was voluntary, which introduces the possibility of social desirability bias—especially on sensitive topics like attitudes toward disability and self-reported practices. The qualitative approach relied on participants’ subjective accounts; key concepts (e.g., “special needs,” “accessibility,” “communication”) might have been understood differently by each person, so caution is needed to ensure consistency in concepts during analysis. Lastly, the absence of observational or simulation-based data restricted our ability to directly observe how volunteers perform their reported practices during actual responses.

## 5. Conclusions

This study identified three interconnected issues: disability is often viewed through narrow, visible categories; volunteers encounter ongoing challenges in identification and communication; and gaps in training and accreditation systematically sustain these limitations. The importance of these findings lies in demonstrating that the constraints are not only individual but also structural, rooted in training curricula, organizational routines, and broader sociocultural perceptions of disability.

Policy and practice should, therefore, incorporate inclusion as a fundamental principle of disaster management. Training curricula for SAR-NGO volunteers need to include disability awareness, inclusive evacuation procedures, and alternative communication strategies as standard components. Accreditation processes conducted by AFAD and INSARAG should require disability-inclusive modules to comply with international human rights and disaster risk reduction standards. Beyond curricula, organizations should establish collaboration with disability advocacy groups to involve people with disabilities as active partners in planning, drills, and evaluation. These steps can enhance both operational capacity and the cultural narratives that currently promote exclusionary practices.

### Implications for Future Research

Future studies should examine different regions of Türkiye to understand diverse organizational environments and volunteer profiles. Participatory approaches involving people with disabilities as co-researchers can provide firsthand insights into barriers and needs, improving the validity and relevance of the findings [34,62]. Evaluation through experiments and simulations is essential to assess the effectiveness of updated training modules (e.g., disability awareness, inclusive evacuation, alternative communication) with mixed results (knowledge, attitudes, field performance). Additionally, research on information systems that support pre-intervention assessment and real-time communication about access needs can help operationalize inclusive capacity on a wider scale.

## Figures and Tables

**Table 1:** Participants Characteristics.

Code	Age	Gender	Occupation	Experience	Organization Accredition	Individual Accredition	Trainer Status
Anıl	29	Male	Worker	5	AFAD	AFAD	-
Aydın	61	Male	Retired	5	AFAD, INSARAG	-	-
Aylin	43	Female	Child care provider	7	AFAD, INSARAG	AFAD	-
Berk	50	Male	Retired	3	AFAD	AFAD	OBAK
Berkay	35	Male	Shopkeeper	1	AFAD	-	OBAK
Bora	38	Male	Worker	3	AFAD	-	OBAK
Emre	55	Male	Retired	25	AFAD, INSARAG	AFAD, INSARAG	AFAD
Eray	50	Male	Teacher	10	AFAD	AFAD	OBAK
Gülce	23	Female	Officer	5	AFAD	AFAD	-
İbrahim	45	Male	Designer	3	AFAD	AFAD	University
Jale	46	Female	Teacher	2	AFAD	AFAD	-
Metin	47	Male	Retired	15	AFAD, INSARAG	AFAD, INSARAG	AFAD
Murat	39	Male	Worker	3	AFAD	-	OBAK
Musa	47	Male	Officer	10	AFAD	AFAD	University
Orhan	58	Male	Retired	3	AFAD	-	-
Recep	42	Male	Technician	3	AFAD	AFAD	-
Veli	42	Male	CDS	22	AFAD	AFAD	AFAD
Yiğit	25	Male	Accountant	4	AFAD	-	-
Yunus	37	Male	Shopkeeper	2	AFAD	-	OBAK
Yusuf	50	Male	OHSE	11	AFAD, INSARAG	-	-

Note: AFAD = Disaster and Emergency Management Presidency, INSARAG = International Search and Rescue Advisory Group, OBAK = Odunpazarı Municipality Search and Rescue Team, CDS = Civil Defense Specialist, OHSE = Occupational Health and Safety Expert.

## Data Availability

The data presented in this study are available on request from the corresponding author due to privacy or ethical restrictions approved by Anadolu University.
